# Relationship between body dimensions and the diameter of a 4‐strand ACLR graft

**DOI:** 10.1002/jeo2.70329

**Published:** 2025-07-07

**Authors:** WP Yau

**Affiliations:** ^1^ Department of Orthopaedics and Traumatology, School of Clinical Medicine, Li Ka Shing Faculty of Medicine The University of Hong Kong Hong Kong Special Administrative Region P.R. China

**Keywords:** 7 mm, 8 mm, body height, body weight, graft diameter, probability table

## Abstract

**Purpose:**

The objective of this study is to create a probability table outlining the chance of preparing a ‘doubled semitendinosus and doubled gracilis’ (ST×2 + G×2) graft for anterior cruciate ligament reconstruction (ACLR) with minimum diameters of 7 and 8 mm, based on body height (BH) and body weight (BW).

**Methods:**

A retrospective study was conducted on patients who underwent hamstring harvesting between 2008 and 2021. Patients eligible for inclusion were those who (1) were skeletally mature at the time of ACLR, (2) underwent ACLR using a medial hamstring autograft, (3) had both semitendinosus and gracilis tendons harvested and (4) were of the same ethnicity (Chinese). The diameter of the ‘ST×2 + G×2’ graft was recorded in 0.5 mm increments. A probability table was developed using BH intervals of 5 cm and BW increments of 10 kg.

**Results:**

The study included a total of 536 skeletally mature patients, consisting of 444 males and 92 females. The average BH and BW were 171 ± 8 cm and 70.9 ± 12.5 kg, respectively. The average diameter of the ‘ST×2 + G×2’ graft was 8.6 ± 0.9 mm. The preparation of a 4‐strand ACLR graft with a 7 mm diameter was almost always achievable in males (99.1%). In contrast, the successful preparation of a 7 mm graft was not feasible in females weighing less than 40 kg. The likelihood of successfully preparing a 4‐strand graft with an 8 mm diameter was low for males with a BH < 160 cm and a BW < 60 kg. Regardless of BH, the chance of successfully preparing an 8 mm diameter graft in females was high if their BW exceeded 60 kg.

**Conclusion:**

A significant positive association was observed between the diameter of ‘ST×2 + G×2’ and male sex, BH and BW. The likelihood of successfully preparing a 4‐strand graft with a minimum diameter of 8 mm was low for male patients shorter than 160 cm and weighing less than 60 kg. Meanwhile, it is impossible to successfully prepare a 4‐strand ACLR graft with a minimum diameter of 7 mm for female patients weighing less than 40 kg.

**Level of Evidence:**

Level IV.

**Abbreviations**
ACLR: anterior cruciate ligament reconstruction
BH: body height
BW: body weight
G: gracilis
ST: semitendinosus
ST×2 + G×2: doubled semitendinosus and doubled gracilis tendons
TAS: tegner activity scale

## INTRODUCTION

Some surgeons utilize doubled semitendinosus (ST) and doubled gracilis (G) tendons to fashion a 4‐stranded anterior cruciate ligament reconstruction (ACLR) graft (ST×2 + G×2) for single‐bundle ACLR [1]. However, there is significant variability in the diameter of the resulting ACLR graft, ranging from 4.5 to 11 mm [5, 9, 12, 13]. The incidence of ACLR graft failure and the need for revision surgery are recognized to be higher when the graft diameter for single‐bundle ACLR is small [2, 4, 8, 11, 12], although there remains controversy regarding whether the critical threshold is 7 [2, 8] or 8 mm [4, 11, 12]. Patients who undergo hamstring (HS) ACLR with a smaller graft are at a higher risk of experiencing clinical failure within a minimum 2‐year follow‐up period compared to those with a larger graft [9]. Registry data indicates that the relative risk of ACL revision within 2 years is 1.25 times higher [11].

Having a method for predicting patients at risk of receiving a small 4‐stranded HS ACLR graft, regardless of whether it measures 7 or 8 mm, prior to surgery, would be beneficial. Patient‐specific pre‐operative counselling, which includes discussing alternative graft options and the heightened risk of graft failure, as well as enhanced surgical preparation, such as allograft or synthetic graft, can be conducted. Anthropometric data such as body height (BH), body weight (BW) and sex have been linked to the diameter of an HS graft prepared using a doubled ST and doubled G configuration [6, 7, 13]. Shorter BH, lower BW and female sex are associated with an increased likelihood of having a small HS‐ACLR graft [6, 7, 13]. Previous efforts have been made to predict graft diameter using equations derived from regression analysis [10, 13]. However, these methods have not gained widespread acceptance, possibly due to the complexity of the mathematical models [10] and the discrepancy between the predicted value and the actual graft size [5]. It would be useful to develop a simple tool that determines the chance of preparing a 4‐strand ACLR HS graft with a minimum diameter of 7 and 8 mm, considering the BH and BW of each individual patient. This tool can assist surgeons in identifying patients at risk of a small 4‐strand graft. Appropriate counselling can be provided during the consent process. This can include discussions on alternative graft choices.

The aim of this study is to provide a narrative description of the correlation between the diameter of a ‘doubled semitendinosus and doubled gracilis’ (ST×2 + G×2) graft and the patient's BH and BW. We intend to develop a probability table illustrating the chance of preparing an ‘ST×2 + G×2’ HS ACLR graft with minimum diameters of 7 and 8 mm based on these factors.

## METHODS

A retrospective study, utilizing data collected prospectively, was conducted at the author's institute. The study involved patients who underwent medial HS harvesting for ACLR between February 2008 and December 2021. The local ethics committee approved the study and waived the need for informed consent from the participants (Document number: UW 24‐120).

Patients eligible for inclusion in the study were those who (1) were skeletally mature at the time of ACLR, (2) underwent ACLR using a medial HS autograft, (3) had both semitendinosus and gracilis tendons harvested and (4) were of the same ethnicity (Chinese). Patients were excluded if (1) they experienced harvesting complications, such as premature graft rupture, or (2) if there were missing data, including dimensions of the ST and G, BW or BH.

Demographic information, including age, sex, BH, BW, laterality of the injured knee and pre‐injury Tegner activity scale (TAS), was gathered during a pre‐operative assessment clinic one week before the surgery. BH and BW were measured to the nearest centimetre and kilogram, respectively.

The medial HS harvesting and ACLRs were performed by two sports medicine surgeons at the author's institute, including WPY. The tendons were doubled at their midpoint and passed through a graft sizer (Smith and Nephew, Watford, United Kingdom), which measured the graft's diameter in 0.5 mm increments. The diameter of the harvested tendon was recorded as the smallest size on the graft sizer, which allowed the passage of two strands of the respective tendon under maximum manual tension. The graft sizer could measure diameters ranging from 4.5 to 12 mm. All data were prospectively recorded on a standard data collection form.

### Statistics

The dimensions of the ST and G tendons, the diameter of ‘ST×2 + G×2’, and the anthropometric data were reported using descriptive statistics. The association between the anthropometric data and the diameter of ‘ST×2 + G×2’ was analyzed through bivariate correlation. A *p*‐value below 0.05 was deemed statistically significant. The strength of the association was categorized as perfect if the correlation coefficient was 1, strong if it fell between 0.7 and 0.9, moderate if it ranged from 0.4 to 0.6 and weak if it ranged from 0.1 to 0.3.

The percentage of ‘ST×2 + G×2’ grafts with a minimum diameter of 7 and 8 mm was calculated relative to BH and BW. A narrative description of the relationship between the diameter of ‘ST×2 + G×2’ and BH and BW in males and females was presented as a probability table. This table describes the chance of preparing an ‘ST×2 + G×2’ HS ACLR graft with a minimum diameter of 7 and 8 mm based on BH and BW in males and females, respectively. The likelihood of having a graft with the specified minimum diameter was classified as low if it ranged from >0% to 33%, medium if it ranged from >33% to 66% and high if it ranged from >66% to 100%.

## RESULTS

**Figure 1:**
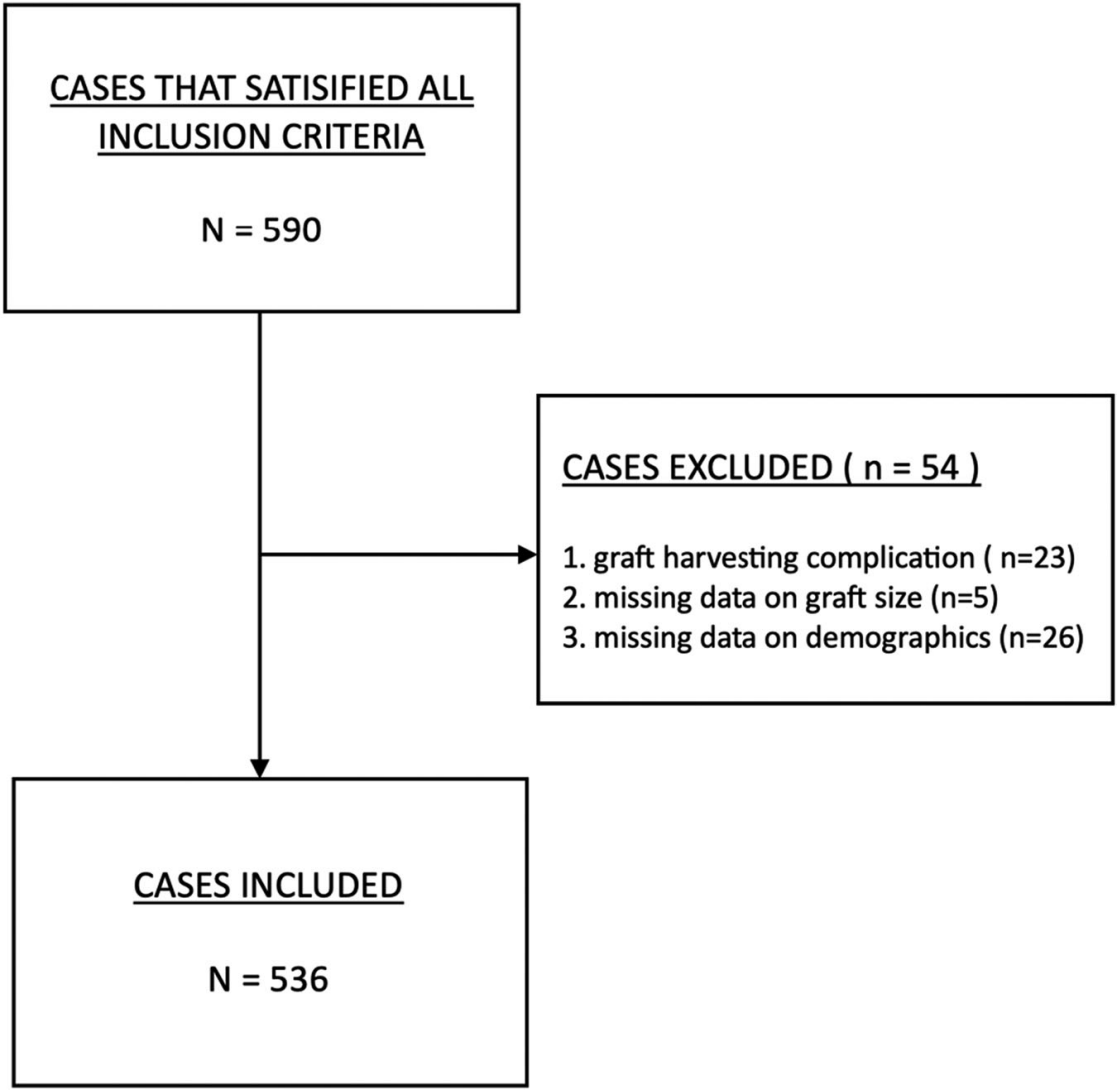
Enrolment of patients.

A total of 536 skeletally mature patients were included in the study, comprising 444 males and 92 females (Figure 1). The average age was 27 ± 7.7 years, ranging from 14 to 52 years. The medial HSs were harvested from the right knee in 282 patients and from the left knee in 254 patients. The average BH and BW were 171 ± 8 cm (ranging from 145 to 190 cm) and 70.9 ± 12.5 kg (ranging from 35 to 125 kg), respectively. The average pre‐operative TAS was 6.5 ± 1.3, ranging from 2 to 9.

The length of the semitendinosus tendon and gracilis tendon was 262 ± 28 mm (ranging from 175 to 380 mm) and 233 ± 28 mm (ranging from 120 to 340 mm), respectively. The diameter of a doubled semitendinosus tendon ranged from 4.5 to 9 mm (mean = 6.6 ± 0.7 mm), while that of a doubled gracilis tendon ranged from 4.5 to 7.5 mm (mean = 5.1 ± 0.7 mm). The diameter of a 4‐strand graft composed of ‘ST×2 + G×2’ was 8.6 ± 0.9 mm, ranging from 5.5 to 11.5 mm.

A significant positive association was observed between the diameter of ‘ST×2 + G×2’ and male sex (*r* = 0.301, *p* < 0.001; weak association), BH (*r* = 0.369, *p* < 0.001; weak to moderate association) and BW (*r* = 0.359, *p* < 0.001; weak to moderate association). There was no association between the diameter of ‘ST×2 + G×2’ with age (*p* = 0.419), knee laterality (*p* = 0.634) and pre‐operative TAS (*p* = 0.227).

The probabilities of a 4‐strand graft in a doubled ST and doubled G configuration having minimum diameters of 8 and 7 mm are presented in Tables 1, 2, 3, 4, respectively.

**Table 1:** Likelihood of having the diameter of an ST×2 + G×2 graft ≥8 mm in males.

BH (cm)	ST×2 + G×2 diameter (mm)	BW: 40–50 (kg)	BW: 50–60 (kg)	BW: 60–70 (kg)	BW: 70–80 (kg)	BW: 80–90 (kg)	BW: 90–100 (kg)	BW: 100–110 (kg)	BW: 110–120 (kg)	BW: 120–130 (kg)	Total
155–160	≥8	0%	L _(25%)_	100%							L _(33%)_
160–165	≥8		H _(88%)_	H _(85%)_	H _(67%)_	100%					H _(85%)_
165–170	≥8		H _(93%)_	H _(74%)_	H _(80%)_	H _(90%)_	100%				H _(81%)_
170–175	≥8		100%	H _(83%)_	H _(89%)_	H _(95%)_	100%	100%	100%	100%	H _(90%)_
175–180	≥8		100%	H _(86%)_	H _(93%)_	H _(84%)_	100%	100%			H _(90%)_
180–185	≥8			H _(80%)_	H _(97%)_	H _(94%)_	100%	100%	100%		H _(95%)_
185–190	≥8			100%	100%	100%	100%		100%		100%
190–195	≥8					100%					100%
TOTAL	≥8	0%	H _(87%)_	H _(81%)_	H _(90%)_	H _(92%)_	100%	100%	100%	100%	H _(87.6%)_

*Note*: L: low chance (>0% to 33%); M: medium chance (>33% to 66%); H: high chance (>66% to <100%).

Abbreviations: BH, body height; BW, body weight; ST×2 + G×2, doubled semitendinosus and doubled gracilis tendons.

**Table 2:** Likelihood of having the diameter of an ST×2 + G×2 graft ≥8 mm in females.

BH (cm)	ST×2 + G×2 diameter (mm)	BW: 30–40 (kg)	BW: 40–50 (kg)	BW: 50–60 (kg)	BW: 60–70 (kg)	BW: 70–80 (kg)	BW: 80–90 (kg)	BW: 90–100 (kg)	Total
145–150	≥8	0%		100%					M _(50%)_
150–155	≥8		0%	H _(75%)_					M _(38%)_
155–160	≥8		M _(40%)_	L _(25%)_	H _(67%)_	100%			M _(39%)_
160–165	≥8		100%	M _(58%)_	H _(70%)_		100%		H _(67%)_
165–170	≥8			L _(33%)_	H _(78%)_	100%	100%	100%	H _(80%)_
170–175	≥8			100%		100%		100%	100%
175–180	≥8				100%	100%			100%
Total	≥8	0%	L _(30%)_	M _(45%)_	H _(74%)_	100%	100%	100%	M _(60%)_

*Note*: L: low chance (>0% to 33%); M: medium chance (>33% to 66%); H: high chance (>66% to <100%).

Abbreviations: BH, body height; BW, body weight; ST×2 + G×2, doubled semitendinosus and doubled gracilis tendons.

**Table 3:** Likelihood of having the diameter of an ST×2 + G×2 graft ≥7 mm in males.

BH (cm)	ST×2 + G×2 diameter (mm)	BW: 40–50 (kg)	BW: 50–60 (kg)	BW: 60–70 (kg)	BW: 70–80 (kg)	BW: 80–90 (kg)	BW: 90–100 (kg)	BW: 100–110 (kg)	BW: 110–120 (kg)	BW: 120–130 (kg)	Total
155–160	≥7	100%	H _(75%)_	100%							H _(83%)_
160–165	≥7		H _(88%)_	100%	100%	100%					H _(93%)_
165–170	≥7		100%	100%	100%	100%	100%				100%
170–175	≥7		100%	100%	H _(98%)_	H _(95%)_	100%	100%	100%	100%	H _(99%)_
175–180	≥7		100%	100%	100%	100%	100%	100%			100%
180–185	≥7			100%	100%	100%	100%	100%	100%		100%
185–190	≥7			100%	100%	100%	100%		100%		100%
190–195	≥7					100%					100%
Total	≥7	100%	H _(95%)_	100%	H _(99%)_	H _(99%)_	100%	100%	100%	100%	H _(99%)_

*Note*: L: low chance (>0% to 33%); M: medium chance (>33% to 66%); H: high chance (>66% to <100%).

Abbreviations: BH, body height; BW, body weight; ST×2 + G×2, doubled semitendinosus and doubled gracilis tendons.

**Table 4:** Likelihood of having the diameter of an ST×2 + G×2 graft ≥7 mm in females.

BH (cm)	ST×2 + G×2 diameter (mm)	BW: 30–40 (kg)	BW: 40–50 (kg)	BW: 50–60 (kg)	BW: 60–70 (kg)	BW: 70–80 (kg)	BW: 80–90 (kg)	BW: 90–100 (kg)	Total
145–150	≥7	0%		100%					M _(50%)_
150–155	≥7		H _(75%)_	100%					H _(88%)_
155–160	≥7		M _(60%)_	H _(90%)_	100%	100%			H _(87%)_
160–165	≥7		100%	H _(92%)_	100%		100%		H _(96%)_
165–170	≥7			100%	100%	100%	100%	100%	100%
170–175	≥7			100%		100%		100%	100%
175–180	≥7				100%	100%			100%
Total	≥7	0%	H _(70%)_	H _(93%)_	100%	100%	100%	100%	H _(93%)_

*Note*: L: low chance (>0% to 33%); M: medium chance (>33% to 66%); H: high chance (>66% to <100%).

Abbreviations: BH, body height; BW, body weight; ST×2 + G×2, doubled semitendinosus and doubled gracilis tendons.

In the current study, the BW and BH of skeletally mature males ranged from 40 to 130 kg and 155 to 195 cm, respectively. It was almost always feasible to prepare a 4‐strand ACLR graft with a minimum diameter of 7 mm in a skeletally mature male (99.1%) (Table 3). The corresponding chance for a graft ≥8 mm was 87.6%. The likelihood of successfully preparing a 4‐strand graft with a minimum diameter of 8 mm was low for male patients who were less than 160 cm in height and less than 60 kg (Table 1).

In the current cohort, the BW and BH of a skeletally mature female ranged from 30 to 100 kg and 145 to 180 cm, respectively. The chances of successfully preparing a 4‐strand ACLR graft with a minimum diameter of 7 and 8 mm were 93% and 60%, respectively. The successful preparation of a 4‐strand ACLR graft with a minimum diameter of 7 mm was not achievable in a female patient weighing less than 40 kg. Irrespective of BH, the probability of successfully preparing a graft with a minimum diameter of 8 mm was high in a female if her BW exceeded 60 kg (Tables 2 and 4).

## DISCUSSION

The most important findings of this study was that the likelihood of successfully preparing a 4‐strand graft with a minimum diameter of 8 mm was low for male patients who were less than 160 cm in height and weighing less than 60 kg, while the successful preparation of a 4‐strand ACLR graft with a minimum diameter of 7 mm was not achievable in a female patient weighing less than 40 kg.

There is a significant variation in the reported incidence of a 4‐strand ACLR graft with a diameter less than 8 mm in the literature (Table 5) [5, 7, 10, 12, 13]. The likelihood of having a 4‐strand ACLR graft diameter of less than 8 mm was 5.8% in a group of 725 Caucasian patients [7]. Conversely, the corresponding percentage in a cohort of 296 Koreans was as high as 77% [9]. The incidence was 43% in studies involving Indian patients [5] and Chinese patients [12]. Regarding the proportion of grafts with a diameter less than 7 mm, aside from the research by Park et al., most studies reported an incidence ranging from 1% to 4% [5, 7, 10, 12, 13]. The probability of patients having a small graft in the current study was similar to the findings reported by Ramkumar et al.

**Table 5:** Published literature on the anthropometric analysis of the diameter of a 4‐strand hamstring anterior cruciate ligament reconstruction graft.

	Total number of patients	Body height (cm)	Body weight (kg)	Rate of ACLR graft diameter of less than 7 mm (%)	Rate of ACLR graft diameter of less than 8 mm (%)
Goyal et al. [5]	160	169 ± 7	69.2 ± 11.7	4%	43.1%
Janssen et al. [7]	725	177 ± 9	76.5 ± 13.4	n/a	5.8%
Park et al. [9]	296	171±8	72.1 ± 12.2	26.3%	76.9%
Ramkumar, et al. [10]	1681	173 ± 10	80.1 ± 18.6	1%	16.9%
Tang et al. [12]	394	n/a	n/a	4.3%	42.9%
Tuman et al. [13]	106	172 ± 9	75.4 ± 14.9	2%	48.2%
Current study	562	171 ± 8	70.9 ± 12.5	2.1%	17.2%

Abbreviations: ACLR, anterior cruciate ligament reconstruction graft; n/a, not available.

It is well‐established that BH, BW and sex correlate with the diameter of a 4‐strand ACLR graft [5, 7, 10, 12, 13]. Additionally, it is known that the risk of graft failure increases when the graft diameter is smaller, irrespective of whether the critical threshold is defined as 7 [2, 8] or 8 mm [4, 11, 12]. However, the size of the HS graft is only determined after it has been harvested and measured. Hence, a method that could identify patients at risk of having a 4‐strand ACLR HS graft smaller than the critical threshold before harvesting the medial HS would be beneficial. A descriptive narrative outlining the relationship between key anthropometric data, such as BH, BW and the likelihood of successfully preparing a 4‐strand ACLR graft with minimum diameters of 7 and 8 mm, provides a straightforward method for surgeons to predict the likelihood of obtaining a sufficiently sized graft in patients. This method is devoid of complex mathematical manipulations and is likely applicable to various data sets. According to the findings of the current study, the diameter of a 4‐strand ACLR HS graft for a male patient shorter than 160 cm and weighing less than 60 kg is likely to be smaller than 8 mm. Meanwhile, for a female patient weighing less than 40 kg, the graft diameter is consistently smaller than 7 mm. Patients with these body dimensions should be advised against using an ipsilateral medial HS autograft as the sole source of graft for ACLR. Alternative graft choices, such as bone‐patellar tendon‐bone graft or quadriceps tendon graft, should be recommended.

The current study used data collected from patients of the same ethnicity. Chiang et al. reported differences in the dimensions of HS tendons among patients of various ethnicities [3]. It remains unclear whether the disparities noted by Chiang et al. are due to ethnic variations or differences in anthropometric data, such as BH and BW, across different populations. Further research is needed to elucidate the relationship between ethnicity and HS tendon dimensions.

### Limitation

The first limitation of this study is its retrospective nature, which makes it more susceptible to bias despite the prospective collection of all data. Second, the study was carried out using data from patients of a single race. It is uncertain whether the findings from this study can be generalized to patients of other ethnicities. Third, the study included a total of 444 males and 92 females, leading to an uneven sex distribution that could introduce bias. Another limitation of this study is the absence of evidence suggesting that the rupture rate of a 7 mm or even a 6 mm diameter ACLR graft is higher among a subgroup of patients who are short and thin. In addition, the current study is a narrative report. Other than the descriptive statistics concerning the central frequency and spread of the data, the only statistical analysis performed involves testing the association between the diameter of the 4‐strand ACLR graft and various anthropometric factors. The lack of robustness in the analysis represents a major limitation of this study. The other major limitations of this study include the lack of novelty and the lack of superiority when compared to previous studies. Furthermore, the current study does not provide information on the use of tripled or quadrupled semitendinosus tendons, which has become a common practice for many surgeons nowadays. Finally, the classification of the likelihood of successful preparation of a ST×2 + G×2 graft with a minimum diameter of 7 or 8 mm into low, medium and high categories using the ranges >0% to 33%, >33% to 66% and >66% to <100% is arbitrary and may not be universally accepted.

## CONCLUSION

A significant positive association was observed between the diameter of ‘ST×2 + G×2’ and male sex, BH and BW. The likelihood of successfully preparing a 4‐strand graft with a minimum diameter of 8 mm was low for male patients shorter than 160 cm and weighing less than 60 kg. Meanwhile, it is impossible to successfully prepare a 4‐strand ACLR graft with a minimum diameter of 7 mm for female patients weighing less than 40 kg.

## AUTHOR CONTRIBUTIONS

The authors contributed to the study conception and design. Material preparation, data collection and analysis were performed by WP Yau. The first draft of the manuscript was written by WP Yau commented on previous versions of the manuscript. The author read and approved the final manuscript.

## CONFLICT OF INTEREST STATEMENT

The author declares no conflicts of interest.

## ETHICS STATEMENT

The current study involves human participants. The local ethics committee (Institutional Review Board of the University of Hong Kong/Hospital Authority Hong Kong West Cluster) approved and monitored the current study (Document number: UW 24‐120). The local ethics committee (Institutional Review Board of the University of Hong Kong/Hospital Authority Hong Kong West Cluster) waived the requirement for obtaining informed consent from the participants (Document number: UW 24‐120).

## Data Availability

The data of this study are available from author upon reasonable request and with permission of the local ethics committee.
